# Dieulafoy's Lesion: Decade-Long Trends in Hospitalizations, Demographic Disparity, and Outcomes

**DOI:** 10.7759/cureus.9170

**Published:** 2020-07-13

**Authors:** Raja Chandra Chakinala, Shantanu Solanki, Khwaja F Haq, Jagmeet Singh, Harshil Shah, Dhanshree Solanki, Asim Kichloo, Khwaja S Haq, Azam H Burney, Shanza Waqar, Manasee Vyas, Savneek Chugh, Christopher Nabors

**Affiliations:** 1 Internal Medicine, Independent Researcher, Sayre, USA; 2 Hospital-Based Medicine, Geisinger Commonwealth School of Medicine, Scranton, USA; 3 Gastroenterology, Henry Ford Hospital, Detroit, USA; 4 Nephrology, Geisinger Commonwealth School of Medicine, Scranton, USA; 5 Hospital Administration, Rutgers University, New Brunswick, USA; 6 Internal Medicine, Central Michigan University, Saginaw, USA; 7 Medicine, Kingsbrook Jewish Medical Center, Brooklyn, USA; 8 Medicine, MedCare Clinics, St. Catharines, CAN; 9 Medicine, Mahatma Gandhi Institute of Health Sciences, Mumbai, IND; 10 Nephrology, Westchester Medical Center, Valhalla, USA; 11 Internal Medicine, Westchester Medical Center, Valhalla, USA

**Keywords:** dieulafoy's lesion, gastrointestinal disorders, epidemiology, endoscopy, gastrointestinal bleeding, current trends

## Abstract

Background

Dieulafoy's lesion is a relatively rare, but potentially life-threatening, condition where a tortuous arteriole, most commonly in the stomach, may bleed and lead to significant gastrointestinal hemorrhage. Limited epidemiological data exist on patient characteristics and the annual number of hospitalizations associated with such lesions. The aim of our study is to determine the inpatient burden of Dieulafoy’s lesion.

Methods

We analyzed the National Inpatient Sample (NIS) database for all subjects with a discharge diagnosis of Dieulafoy's lesion of the stomach, duodenum, and colon using International Classification of Diseases, 9th Revision, Clinical Modification (ICD-9-CM) codes 537.84 and 569.86 as the primary or secondary diagnosis during the period from 2002 to 2011. Statistical significance of variation in the number of hospital discharges and demographics during the study period was achieved using the Cochrane-Armitage trend test.

Results

In 2002, there were 1,071 admissions with a discharge diagnosis of Dieulafoy's lesion as compared to 7,414 in 2011 (p < 0.0001). Dieulafoy's lesion was found to be most common in the age group of 65-79 years (p < 0.0001). Overall, it was found to be more common in males as compared to females (p = 0.0261). The white race was most commonly affected amongst all the races. The average cost of care per hospitalization increased from $14,992 in 2002 to $25,594 in 2011 (p < 0.0001).

Conclusion

There has been a steady rise in the number of inpatient admissions with Dieulafoy's lesions. Advances in diagnostic techniques likely play a key role in the higher detection rates along with the possible involvement of other unknown factors. Men, in the age group of 65 to 79 years, and Whites were found to have significantly higher admission rates than all other groups, with a significant increase in the cost of care.

## Introduction

Dieulafoy’s lesion (DL) is a dilated submucosal artery that erodes the overlying intestinal mucosa in the absence of an underlying ulcer, aneurysm, or intrinsic mural abnormality [1]. DL is most commonly located in the stomach, but it is also reported to occur throughout the gastrointestinal (GI) tract [1]. It is underdiagnosed and accounts for 1%-2% of cases of acute GI bleeding [2]. Patients are typically asymptomatic until they present with GI bleeding, which may manifest as hematemesis, hematochezia, or melena. It's anatomy and conspicuous nature often pose a diagnostic challenge. Even after appropriate investigation, the lesion may remain obscure and cause a life-threatening hemorrhage, as the bleeding can be massive and recurrent [3].

The under-recognition contributes to its increased morbidity and previously reported high mortality associated with it. Given its impact on patient outcomes and healthcare costs, it is important to understand its epidemiology, patient demographics, associated comorbidities, and hospitalization trends. Currently, there are is no nationwide analysis highlighting the economic burden of DL in the US. The aim of our study is to determine the inpatient burden of DL in the US.

## Materials and methods

Source of data

The National Inpatient Sample (NIS), designed by the Agency for Healthcare Research and Quality (AHRQ), is the largest all-payer inpatient database in the U.S. Data are compiled yearly and contain discharge information from over 1200 hospitals located across 45 states in the U.S. The NIS was designed to approximate a 20% stratified sample of community hospitals in the country and provides sampling weights to calculate national estimates [4]. The NIS contains information included in a typical discharge summary, with safeguards in place to protect the privacy of individual patients, physicians, and hospitals. Each individual hospitalization is de-identified and maintained in the NIS as a unique entry, with one primary discharge diagnosis and approximately 24 secondary diagnoses during that hospitalization. Each entry also carries information on demographic details, insurance status, comorbidities, primary/secondary procedures, hospitalization outcomes, length of stay, and cost of care. The internal validity of the database is guaranteed by annual data quality assessments of the sample. Moreover, comparisons with data sources like the American Hospital Association (AHA) Annual Survey of Hospitals, National Hospital Discharge Survey from the National Center for Health Statistics, and MedPAR inpatient data from the Centers for Medicare and Medicaid Services strengthen the external validity of the sample [5-6].

Study design

This is a retrospective cohort study in which we queried the NIS database from the year 2002 to 2011 to identify all the hospitalizations with DL. We extracted data for all the hospitalizations from 2002 to 2011 with a primary or secondary diagnosis of DL, which, in turn, was identified with ICD-9 codes 537.84 (stomach and duodenum) and 569.86 (colon). Patients aged less than 18 years were excluded. Also, hospitalizations with missing information related to age, gender, admission/discharge date, in-hospital mortality status, demographics, and comorbidities were excluded as seen in previous well-designed studies [7]. To calculate the estimated cost of hospitalizations, NIS data were merged with the cost-to-charge ratio (CCR) files available from the Healthcare Cost and Utilization Project (HCUP). We estimated the cost of each inpatient stay by multiplying the total hospital charge with a cost-to-charge ratio.

Variables and statistical analysis

SAS 9.4 (SAS Institute Inc., Cary, North Carolina, USA) was utilized for complex statistical analyses. Since NIS represents a 20% stratified random sample of U.S. hospitals, analyses were performed using hospital-level discharge weights provided by the NIS to obtain national estimates of hospitalizations. The frequency of paralytic ileus-related hospitalizations was calculated for each year. We calculated hospitalizations related to Dieulafoy’s lesions per 1-million U.S. population by dividing yearly hospitalizations by 20% of the U.S. census population more than 18 years of age for that year [8]. The Cochrane-Armitage trend test was used to calculate trends in categorical variables [9]. The Wilcoxon rank-sum test was used to assess continuous variables. These hospitalizations were also calculated in subgroups of age (18-34, 35-49, 50-64, 65-79, and >80 years), gender, race (White, Black, Hispanic, and Others), insurance status (Medicare/Medicaid, private insurance, and self-pay/other), hospital location in different U.S. regions (Northeast, Midwest, South, and West), bed size of the hospital (small, medium, and large), and teaching status of the hospital (urban teaching, urban non-teaching, and rural). According to AHRQ, a hospital is considered to be a teaching hospital if it is: a) an AMA-approved residency program, b) a member of the Council of Teaching Hospitals or c) a hospital with a full-time resident-to-bed ratio more than 0.25 [10].

## Results

Demographics

Patient characteristics are summarized in Table 1.

**Table 1:** Baseline characteristics of Dieulafoy’s lesion hospitalizations AHRQ: Agency for Healthcare Research and Quality

Year	2002	2003	2004	2005	2006	2007	2008	2009	2010	2011	Average	P-VAL
Number of obs. (n)	1,071	4,972	5,175	4,940	5,303	5,417	6,231	6,380	6,685	7,414	5,359	<0.0001
Age in years (%)												
18-34	3.6	2.5	3.3	3.2	2.7	2.6	2.6	3.2	2.9	2.4	2.8	0.07
35-49	8.1	7.1	7.7	7.9	7.1	8.8	9.5	7.8	7.3	7.7	7.9	0.24
50-64	16.6	19.0	19.3	20.7	21.3	23.9	22.4	22.0	21.6	23.4	21.5	<0.0001
65-79	41.9	43.1	40.5	37.7	35.5	37.4	36.9	39.3	37.8	36.4	38.2	<0.0001
>=80	29.4	28.0	29.1	30.1	33.2	27.3	28.3	27.6	30.2	29.8	29.3	0.4173
Gender (%)												
Male	52.4	55.3	52.9	54.7	55.6	60.0	57.0	56.3	56.0	54.4	55.7	0.0261
Female	47.6	44.7	47.0	45.2	44.4	40.0	43.1	43.7	44.0	45.6	44.2	
Race (%)												
White	49.2	57.3	56.2	55.0	57.2	51.8	57.9	64.5	66.9	67.5	59.8	0.14
Black	6.1	9.1	8.4	8.8	7.5	9.7	8.8	10.0	11.2	10.6	9.4	0.0748
Hispanic	8.2	6.0	6.5	5.0	6.1	6.9	6.7	6.3	6.5	7.1	6.4	0.0043
Others	4.9	3.3	3.9	3.2	3.8	4.4	5.3	3.9	5.5	5.2	4.4	0.0008
Region (%)												
Northeast	17.6	19.7	20.4	21.9	23.1	20.3	20.4	20.0	20.1	20.3	20.6	0.2
Midwest	23.1	22.8	20.7	21.8	24.0	24.6	23.3	22.8	22.1	23.6	22.9	0.0372
South	38.0	38.9	39.5	36.7	32.3	32.5	36.0	39.0	39.2	37.8	37.0	0.1358
West	21.3	18.6	19.4	19.7	20.7	22.7	20.3	18.1	18.6	18.4	19.6	0.0058
Location (%)												
Rural	12.5	12.5	9.7	8.8	7.0	9.7	10.4	9.2	9.8	8.3	9.5	<0.0001
Urban nonteaching	48.2	46.8	46.8	50.2	44.1	44.9	44.5	42.9	41.2	39.3	44.2	<0.0001
Urban teaching	39.3	40.7	43.5	41.0	48.5	45.2	45.1	46.8	47.3	51.5	45.8	<0.0001
Median household income (%)												
Quartile 1	3.2	22.9	22.8	22.8	24.2	25.2	24.9	23.5	26.3	26.4	24.1	<0.0001
Quartile 2	19.9	24.6	29.1	24.4	23.4	23.5	27.5	26.6	24.0	23.9	25.1	0.15
Quartile 3	27.6	27.6	25.1	28.2	26.0	25.3	23.8	25.1	24.0	25.6	25.6	<0.0001
Quartile 4	48.0	22.5	20.9	22.6	24.2	22.8	22.3	22.3	22.1	22.3	23.0	<0.0001
Payment (%)												
Medicare	69.2	69.6	68.7	68.8	68.2	64.1	63.8	66.7	65.6	67.1	66.9	<0.0001
Medicaid	4.4	4.6	4.4	5.6	4.8	6.3	6.0	6.3	7.9	6.6	5.9	<0.0001
Private insurance	20.7	19.4	20.7	20.0	20.7	23.0	23.1	19.7	20.1	20.8	20.8	0.16
Others (includes self-pay)	5.7	6.2	6.1	5.7	6.3	6.6	7.1	7.3	6.2	5.1	6.3	0.38
Bed size												
Small	11.6	9.2	11.0	11.0	14.3	10.1	8.9	9.8	9.3	11.0	10.5	0.0373
Medium	21.2	27.5	23.2	26.3	24.4	28.2	21.9	22.7	22.3	24.9	24.4	0.0003
Large	67.2	63.4	65.8	62.7	61.0	61.5	69.3	66.5	66.7	63.2	64.7	<0.0001
In-hospital mortality (%)	3.7	5.1	4.8	4.3	4.1	4.3	3.9	4.7	4.7	4.2	4.4	<0.0001
Cost of care ($)	14,992	19,378	20,115	17,628	20,530	19,284	20,104	21,206	21,629	25,594	20,046	<0.0001
AHRQ comorbidity measures (%)												
Obesity	0.5	1.9	2.6	2.9	5.3	4.9	5.6	6.4	6.5	8.8	5.2	<0.0001
Hypertension	43.0	45.9	54.1	50.8	60.7	56.0	59.5	62.1	63.7	65.7	58.0	<0.0001
Diabetes mellitus	20.0	22.3	22.4	24.9	24.5	28.3	27.8	28.5	28.6	32.6	26.9	<0.0001
Congestive heart failure	17.3	18.4	15.4	21.0	21.3	19.9	17.9	19.4	22.3	21.2	19.7	<0.0001
Chronic pulmonary disease	20.0	16.9	19.1	19.8	24.0	19.9	18.9	20.2	23.2	22.2	20.6	<0.0001
Peripheral vascular disease	9.1	6.0	8.6	7.7	8.0	9.0	10.5	11.1	11.1	12.5	9.6	<0.0001
Renal failure	25.8	30.6	31.0	36.4	42.2	44.5	45.0	48.8	49.0	50.7	42.5	<0.0001
Neurological disorders	4.3	6.3	7.8	7.5	7.0	8.0	8.9	9.5	9.8	9.7	8.4	<0.0001
Anemia	24.7	23.5	25.4	24.1	28.1	28.9	28.9	31.6	32.9	32.8	71.2	<0.0001
Solid tumor without metastasis	7.5	2.4	1.7	2.2	1.7	2.5	1.7	2.4	2.1	3.1	2.3	0.43
Rheumatological disorders	2.2	2.5	2.7	2.7	2.2	2.6	2.9	3.9	2.7	3.4	2.9	<0.0001
Psychiatric disorders	3.7	6.0	6.2	9.0	8.6	11.4	10.4	11.5	11.7	14.5	10.1	<0.0001
Liver disease	7.1	6.0	7.3	7.5	6.9	8.3	8.7	11.0	8.2	11.0	8.5	<0.0001

**Figure 1:**
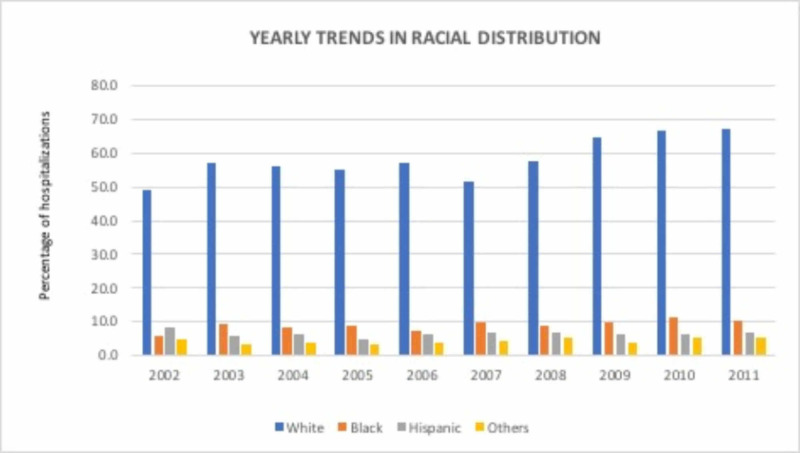
Racial distribution of Dieulafoy’s lesion hospitalizations

**Figure 2:**
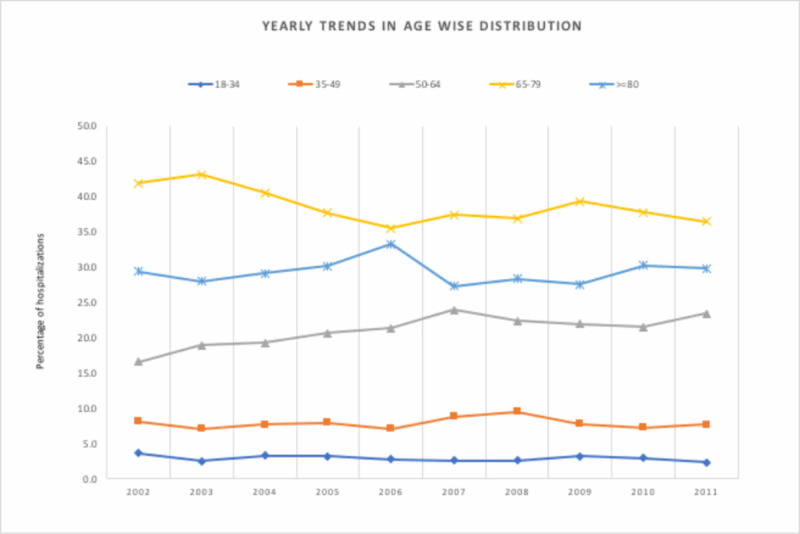
Age-wise distribution of Dieulafoy’s lesion hospitalizations

The number of hospitalizations with a discharge diagnosis of DL increased progressively from 1,071 admissions in 2002 as compared to 7,414 in 2011 (p <0.0001). A total of 53,588 hospitalizations with DL were reported during this time period. These patients were predominantly White (Figure 1) and in the 65 to 74 years age group (Figure 2). Men (55.7%) accounted for a higher number of hospitalizations than women (44.2%) and this ratio remained stable throughout the study period. The highest number of hospitalizations was seen in the South (37.0%) and the lowest was seen in the West (19.6%). Most hospitalizations were seen in urban teaching hospitals (45.8%) followed by urban non-teaching hospitals (44.2%) and rural hospitals (9.5%). Hospitals with a large bed size accounted for 64.7% of the total hospitalizations. Medicare paid for 66.9% of the total hospitalizations whereas Medicaid paid for a mere 5.9% of the hospitalizations.

Trends in hospitalizations

**Figure 3:**
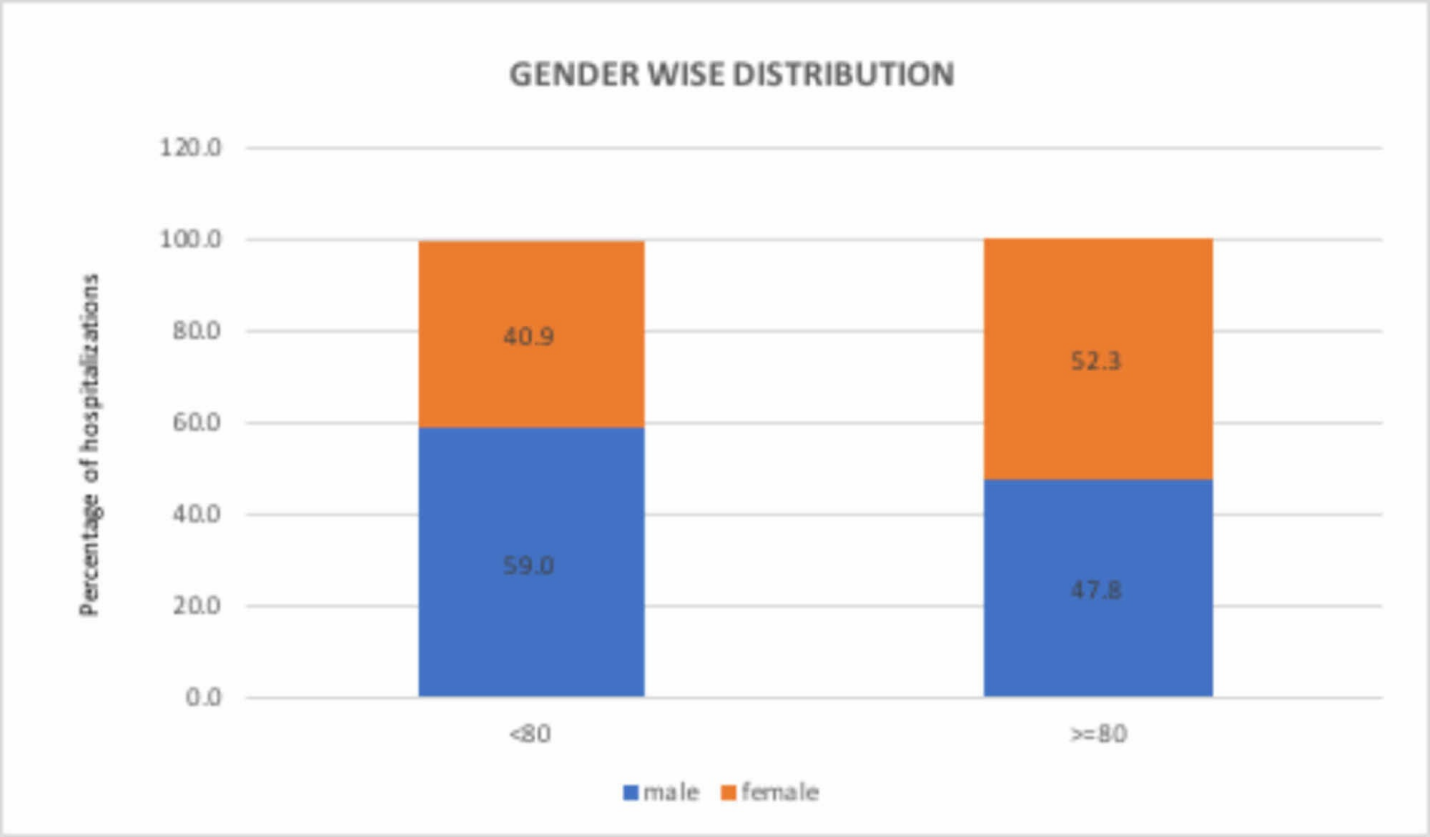
Sensitivity analysis: gender-wise distribution of Dieulafoy’s lesion hospitalizations

The hospitalization rate increased significantly by 524% during the study period, from 25 to 156 per 1-million U.S. population per year from 2002 to 2011 (p <0.0001; Table 2). The hospitalization rate increased significantly for the age group 50-64 (p < 0.0001; Table 2) and was higher in White men throughout the study period. The percent increase in hospitalizations was higher in men compared to women (550% versus 499%, p <0.0001; Table 2) and for Blacks as compared with Whites (992% versus 758%, p <0.0001; Table 2). The Northeast witnessed the highest percent increase in hospitalization and the West witnessed the lowest rate (622% vs 439%, p <0.0001; Table 2). The respective mean ages (years) for the study sample were: overall 69.3 ± SD 33.6 days, for men 67.7 ± SD 33.8 days, and for women 71.5 ± SD 32.6 days for women. The male-to-female ratio differed for age groups <80 years versus >=80 years. In a sensitivity analysis, that ratio was 59.0% vs 40.9% in patients <80 years of age whereas it was 47.8% vs 52.3 in patients >=80 years of age (Figure 3).

**Table 2:** Dieulafoy’s lesion hospitalizations (%) per hundred thousand hospitalizations

YEAR	2002	2003	2004	2005	2006	2007	2008	2009	2010	2011	Average	Percent Increase	P-value
Per Million	25	115	118	111	118	119	135	137	143	156	118	525.6	<0.0001
Age in years													
18-34	1	3	4	4	3	3	4	4	4	4	3	313.1	<0.0001
35-49	2	8	9	9	8	11	13	11	10	12	9	495.3	<0.0001
50-64	4	22	23	23	25	28	30	30	31	37	25	781.4	<0.0001
65-79	10	49	48	42	42	45	50	54	54	57	45	444.1	<0.0001
>=80	7	32	34	34	39	33	38	38	43	46	34	534.0	<0.0001
Gender													
Male	13	63	62	61	66	72	77	77	80	85	66	549.6	<0.0001
Female	12	51	55	50	52	48	58	60	63	71	52	499.1	<0.0001
Race													
White	12	66	66	61	68	62	78	88	95	105	70	757.9	<0.0001
Black	2	10	10	10	9	12	12	14	16	17	11	992.4	<0.0001
Hispanic	2	7	8	6	7	8	9	9	9	11	8	438.4	<0.0001
Others	1	4	5	4	4	5	7	5	8	8	5	560.5	<0.0001
Region													
Northeast	4	23	24	24	27	24	28	27	29	32	24	622.0	<0.0001
Midwest	6	26	24	24	28	29	31	31	31	37	27	539.9	<0.0001
South	9	45	47	41	38	39	49	54	56	59	44	520.9	<0.0001
West	5	21	23	22	24	27	28	25	27	29	23	439.1	<0.0001
Location													
Rural	3	14	11	10	8	12	14	13	14	13	11	315.6	<0.0001
Urban nonteaching	12	54	55	56	52	54	60	59	59	61	52	410.2	<0.0001
Urban teaching	10	47	51	46	57	54	61	64	67	80	54	720.0	<0.0001
Median Household Income													
Quartile 1	1	26	27	25	29	30	34	32	37	41	28	5122.0	<0.0001
Quartile 2	5	28	34	27	28	28	37	36	34	37	30	649.5	<0.0001
Quartile 3	7	32	30	31	31	30	32	34	34	40	30	480.7	<0.0001
Quartile 4	12	26	25	25	29	27	30	31	31	35	27	191.2	<0.0001
Payment													
Medicare	17	80	81	77	80	76	86	91	93	105	79	506.8	<0.0001
Medicaid	1	5	5	6	6	7	8	9	11	10	7	849.0	<0.0001
Private insurance	5	22	24	22	24	27	31	27	29	32	25	527.7	<0.0001
Others (includes self-pay)	1	7	7	6	7	8	10	10	9	8	7	458.0	<0.0001
Bed size													
Small	3	11	13	12	17	12	12	13	13	17	12	495.0	<0.0001
Medium	5	31	27	29	29	34	30	31	32	39	29	634.5	<0.0001
Large	17	73	78	70	72	73	94	91	95	99	76	488.4	<0.0001

Length of stay and cost of care

Median LOS was five days (interquartile range 3-9 days). There was a significant increase in the mean length of stay from 6.7 days to 8.7 days (28.6% increase; p = 0.018). The mean cost of care (adjusted for inflation) increased from $14,992 per hospitalization in 2002 to $25,594 in 2011 (70.7% increase; p <0.0001) (Table 1). The total cost of all such hospitalizations increased from $16 million in 2002 to $189 million in 2011.

AHRQ co-morbidities

**Figure 4:**
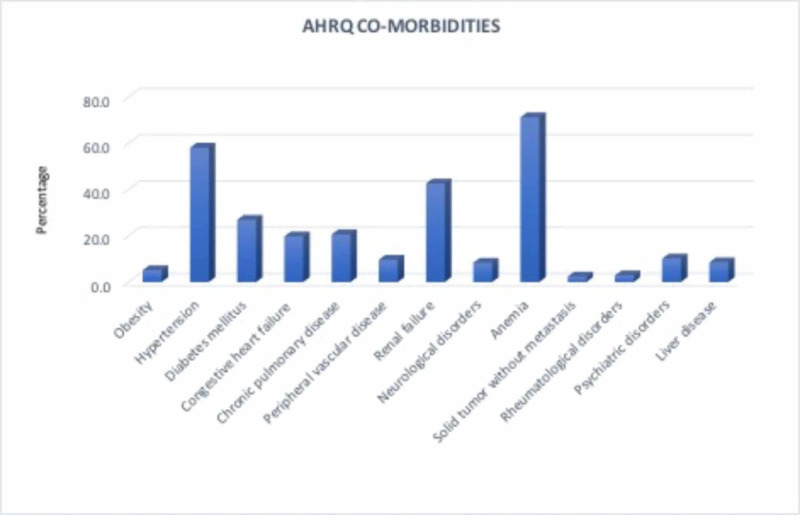
AHRQ co-morbidity measures AHRQ: Agency for Healthcare Research and Quality

The most frequent coexisting conditions in these patients were anemia (71.2%), hypertension (58.0%), and renal failure (42.5%) (Figure 4). The prevalence of several other comorbidities also increased significantly as depicted in Table 1. It is very important to note obesity was identified as a comorbidity in 0.5% of the hospitalizations in 2002 whereas the percent contribution increased to 8.8% in 2011 accounting for a staggering 18-fold increase.

All-cause inpatient mortality

**Figure 5:**
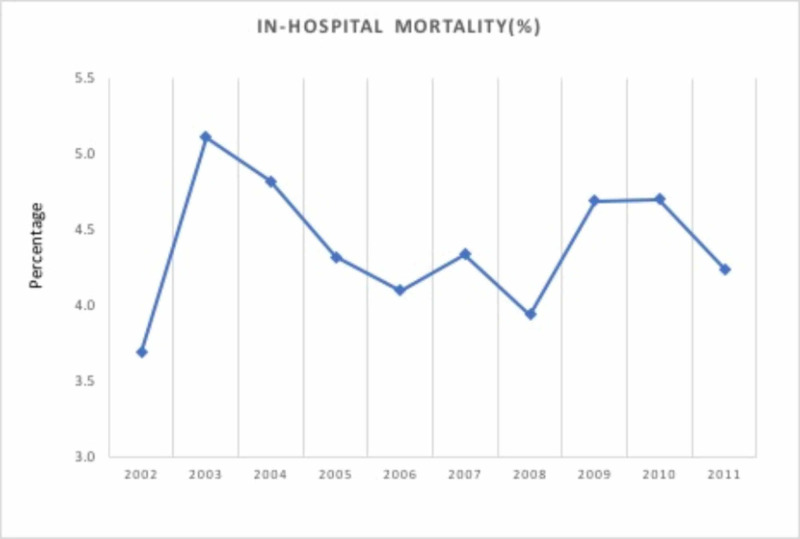
In-hospital mortality for Dieulafoy’s lesion hospitalizations

Overall in-hospital all-cause mortality associated with these hospitalizations was 4.4%. It increased significantly from 3.7% in 2002 to 4.2% in 2011 (percent increase, 14.9%; p <0.0001) (Figure 5). The mortality rate was highest in the ≥80-year age group at 5.2%. The mortality rate was higher in males (4.8%), Blacks (6.3%), and in urban teaching hospitals (5.5%) with a large bed size (4.7%). Also, the mortality rate was noted to be highest in hospitalizations paid by Medicaid (7.1%) (Table 3).

**Table 3:** All-cause in-hospital mortality (%) for Dieulafoy’s lesion hospitalizations *percentage change couldn’t be calculated for groups with value “zero”

YEAR	2002	2003	2004	2005	2006	2007	2008	2009	2010	2011	Average	Percent Change	P-value
Overall (%)	3.7	5.1	4.8	4.3	4.1	4.3	3.9	4.7	4.7	4.2	4.4	14.9	<0.0001
Age in years (%)													
18-34	0.0	0.0	0.0	3.2	0.0	7.5	0.0	0.0	5.6	0.0	1.8	*	0.07
35-49	0.0	5.5	4.7	6.0	2.5	0.9	1.9	7.7	2.5	5.1	3.9	*	0.18
50-64	2.8	3.0	4.9	4.8	2.6	3.9	4.2	3.8	6.1	6.0	4.5	116.7	0.001
65-79	4.4	5.5	4.2	4.0	5.2	4.6	2.7	4.8	3.9	3.4	4.2	-23.6	0.50
>=80	4.7	6.4	6.2	4.1	4.6	5.1	6.5	4.9	5.2	4.1	5.2	-13.0	0.00
Gender (%)													
Male	4.3	5.2	5.8	3.6	3.9	4.9	3.8	5.7	4.7	5.0	4.8	18.1	0.049
Female	3.1	5.0	3.7	5.2	4.3	3.4	4.1	3.4	4.7	3.3	4.1	7.5	
Race (%)													
White	3.8	4.6	5.4	3.5	3.6	4.2	4.0	5.1	4.5	4.2	4.4	11.1	0.08
Black	7.9	8.0	4.2	6.8	6.3	5.3	6.7	5.4	8.1	5.4	6.3	-31.4	0.04
Hispanic	6.3	7.1	2.6	9.7	1.6	7.7	3.5	5.3	5.6	5.3	5.3	-15.9	0.12
Others	0.0	6.4	5.1	9.5	2.5	2.3	6.4	2.0	1.5	4.1	4.0	*	0.06
Region (%)													
Northeast	5.53	3.4	6.3	4.9	5.5	4.2	4.0	5.6	5.3	5.1	5.0	-7.4	0.12
Midwest	6.1	4.3	5.8	4.1	4.1	3.6	2.4	5.8	3.5	2.6	4.0	-56.7	0.0002
South	1.2	4.0	3.3	4.5	3.8	4.8	4.9	3.5	5.7	4.5	4.3	278.3	0.11
West	3.99	10.1	5.4	3.5	3.1	4.6	3.9	4.9	3.4	4.7	4.7	18.3	0.16
Location (%)													
Rural	0.0	3.6	2.3	5.5	4.1	3.9	2.1	0.0	5.8	1.6	3.0	*	0.0089
Urban nonteaching	2.9	4.6	5.0	2.2	4.3	4.2	2.5	4.3	2.7	3.7	3.7	26.1	<0.0001
Urban teaching	5.8	6.2	5.2	6.6	3.9	4.6	5.8	6.1	6.2	4.9	5.5	-15.5	<0.0001
Median household income (%)													
Quartile 1	0.0	4.2	4.0	6.5	4.1	3.6	5.4	4.6	6.3	4.3	4.8	*	0.05
Quartile 2	2.3	6.5	4.4	4.0	4.7	6.7	4.2	4.4	4.8	4.0	4.7	72.5	0.05
Quartile 3	5.3	5.7	3.2	2.6	5.2	3.9	2.8	5.5	5.4	5.0	4.4	-5.1	0.05
Quartile 4	3.7	3.9	8.2	4.6	2.8	3.3	3.5	4.0	1.7	3.5	3.8	-5.4	0.05
Payment (%)													
Medicare	4.0	6.2	4.8	4.0	5.1	4.7	4.4	5.0	4.4	3.7	4.6	-9.2	0.11
Medicaid	0.0	6.5	4.4	14.0	2.0	5.5	7.8	4.8	11.9	5.4	7.1	*	0.01
Private insurance	4.4	2.1	2.7	3.1	2.2	4.0	1.3	3.1	3.2	5.9	3.2	35.2	0.0001
Others (includes self-pay)	0.0	1.4	12.7	3.3	1.5	1.3	4.9	6.4	3.6	2.8	4.2	*	0.06
Bed size													
Small	0.0	2.3	3.7	5.2	3.0	4.9	4.5	2.6	2.8	3.9	3.6	*	0.002
Medium	6.3	4.1	6.5	3.8	2.3	3.7	4.5	4.6	4.0	3.4	4.1	-46.3	<0.0001
Large	3.5	6.0	4.4	4.4	5.1	4.6	3.7	5.1	5.2	4.5	4.7	27.9	<0.0001

## Discussion

To our knowledge, this is the first nationwide analysis of the inpatient burden of DL in the U.S. We found that the total number of hospitalizations with DL increased by nearly six-fold over the study period. It is currently unclear what factors are responsible for the increased prevalence of this condition among hospitalized patients. However, it is possible that several factors may be contributory. Medications like antiplatelet agents, non-steroidal anti-inflammatory drugs (NSAIDs), and anticoagulants, which could potentially cause rupture of submucosal vessels via erosive gastritis, are becoming more commonly used. And conditions such as chronic alcoholism (which may cause mucosal epithelial damage) and cardiovascular disease and renal failure (which may promote the formation of aberrant vessels that are prone to bleeding) are becoming increasingly common [2,11-14]. This may also partly be due to the increased awareness of DL during an endoscopic investigation of upper GI bleeding, resulting in a higher rate of detection.

Anemia, hypertension, and renal failure were found to be the most commonly associated co-morbidities. A similar association of DL with conditions that affect blood coagulation, such as chronic renal failure, cardiovascular disease, neurological, and liver disease, are well-described in the literature [14-18]. As previously suggested by the epidemiological data [2,14-15,17], our analysis also showed the highest incidence per 1 million hospitalizations of DL in the elderly population, i.e. age groups > 50 years with a mean age of 69.3 years. This association is not unexpected as the prevalence of cardiovascular, renal, and other comorbidities is known to increase with age.

Baxter et al. observed DL to be twice as common in males as compared to females [2]. Baettig et al. and Ibanez et al. reported a similar male predominance for DL in their retrospective studies [17-18]. However, Shin et al. reported the occurrence of DL in women two times as often as in men [11]. Our analysis like many others found DL to be more common in males (59%) vs females (40.9%) in the age group <80 years, although the male-female ratio reversed to 47.8% vs 52.3 % in the patients above 80 years of age. While we found higher incidence rates for inpatient DL hospitalization for Whites compared to other racial groups, the increase in hospitalization rate was higher in Blacks compared to Whites during the study period (992.4% versus 757.9%; p <0.0001). The reasons for the difference in occurrence with gender and race is unclear and require further study.

The consequences of DL are significant, as patients are often at risk for re-bleeding, requiring multiple procedures, leading to other complications and prolonged hospital stay. Our findings show that the mean cost per hospitalization increased by 71% from 2002 to 2011. The percent increase in the total annual cost of all such hospitalizations between 2002 to 2011 is 1081% ($16 million in 2002 to $189 million in 2011). To our knowledge, this is the first study to report hospitalization costs in the US for DL.

In early reports, bleeding from DL was associated with high mortality [2,16]. Baxter et al. observed a decrease in mortality in DL patients from 80% to 8.3% [2]. Ibanez et al. reported a mortality rate of 4.9% during hospitalization for DL [17]. We found a mortality rate of 3.7% to 4.2% during the study period. Lower rates of mortality in contemporary studies may stem from a shift from a surgical approach to endoscopic management as the standard modality of management.

A strength of this study is the large sample size made possible through the use of the NIS database. Such a large sample size reduces bias inherent in studies that are confined to a single region or hospital. However, our analysis had a number of significant limitations. Administrative databases are susceptible to errors arising from coding inaccuracies. The diagnosis of DL and the presence of comorbidities were based on the presence of administrative codes. The database did not permit us to determine which hospitalizations assigned a diagnostic code of “DL” were hospitalized for the new diagnosis, as opposed to patients who had a diagnosis of DL in the past. Nor were we able to analyze the influence of medications on the prevalence of DL lesions.

There is a risk that our analysis could underestimate the number of hospitalizations with DL each year if an accompanying condition, such as “gastrointestinal bleeding,” is listed as a primary diagnosis, even if a patient’s primary diagnosis was DL. On the other hand, our analysis could also overestimate the number of patients with DL, as NIS considers each hospitalization as a separate entry, and there is no coding method that can separate index cases from readmissions.

Furthermore, the design of the database only allowed us to examine in-hospital characteristics. The study design limits the analysis of long-term follow-up outcomes or health care utilization in outpatient settings or emergency departments.

## Conclusions

In conclusion, our review of hospitalization trends showed a significant increase in the number of DL-related hospitalizations during the study period, along with an increase in the cost of care. The mortality rate associated with DL stayed low. Further studies are needed to identify factors contributing to the overall increase in the rates of DL and to permit the development of more effective diagnostic and treatment strategies for this entity.
